# Brain responses to odor mixtures with sub-threshold components

**DOI:** 10.3389/fpsyg.2013.00786

**Published:** 2013-10-24

**Authors:** Thomas Hummel, Selda Olgun, Johannes Gerber, Ursula Huchel, Johannes Frasnelli

**Affiliations:** ^1^Department of Otorhinolaryngology, Technical University of Dresden Medical SchoolDresden, Germany; ^2^Department of Neuroradiology, Technical University of Dresden Medical SchoolDresden, Germany; ^3^HenkelDüsseldorf, Germany; ^4^Department of Psychology, Centre de Recherche en Neuropsychologie et Cognition, University of MontrealMontreal, QC, Canada

**Keywords:** fMRI, smell, olfactory, mixing

## Abstract

Although most odorants we encounter in daily life are mixtures of several chemical substances, we still lack significant information on how we perceive and how the brain processes mixtures of odorants. We aimed to investigate the processing of odor mixtures using behavioral measures and functional magnetic resonance imaging (fMRI). The odor mixture contained a target odor (ambroxan) in a concentration at which it could be perceived by half of the subjects (sensitive group); the other half could not perceive the odor (insensitive group). In line with previous findings on multi-component odor mixtures, both groups of subjects were not able to distinguish a complex odor mixture containing or not containing the target odor. However, sensitive subjects had stronger activations than insensitive subjects in chemosensory processing areas such as the insula when exposed to the mixture containing the target odor. Furthermore, the sensitive group exhibited larger brain activations when presented with the odor mixture containing the target odor compared to the odor mixture without the target odor; this difference was smaller, though present for the insensitive group. In conclusion, we show that a target odor presented within a mixture of odors can influence brain activations although on a psychophysical level subjects are not able to distinguish the mixture with and without the target. On the practical side these results suggest that the addition of a certain compound to a mixture of odors may not be detected on a cognitive level; however, this additional odor may significantly change the cerebral processing of this mixture. In this context, FMRI offers unique possibilities to look at the subliminal effects of odors.

## Introduction

Most odors we encounter in daily life arise from the perception of mixtures of several chemical substances. However, while brain responses to single odorous compounds have been relatively well-studied over the last two decades (e.g., Sobel et al., 1998; Savic et al., 2000; Gottfried et al., 2002; Seubert et al., 2012), we still lack significant information on how the brain processes mixtures of odorants.

In one paper brain activations was measured in subjects who were stimulated with either pure odorants or binary mixtures in varying proportions using positron emission tomography (PET). Mixtures activated the cingulate, parietal and superior frontal cortex to a larger extent than the single compounds did. Furthermore, the lateral orbitofrontal cortex (OFC) to be activated strongest after stimulation with binary mixtures of components with the same concentrations (e.g., 50%: 50%), less so by binary mixtures consisting of single compounds in unequal concentrations (e.g., 90%: 10%), and least by single compounds. The anterior OFC on the other hand was activated by mixtures and deactivated by single compounds (Boyle et al., 2009). Further, using a binary mixture of a pleasant and an unpleasant component, some brain regions (e.g., OFC) exhibited activation patterns consistent with the pleasant component whereas activations of other areas (e.g., anterior cingulate) were consistent with the unpleasant component (Grabenhorst et al., 2007). However, although these studies investigated how the brain reacts to mixtures consisting of odorants of different concentrations/valence or of single compounds, it does not yet fully explain the neural basis of odor mixtures perception. For example, we know that subjects are not able to perform better than they would by chance when asked to detect a highly familiar odor within a mixture consisting of 16 different odors (Jinks and Laing, 1999). In fact, we appear to be able to detect a single component within a mixture only if the mixture consists of less than five odorants (Livermore and Laing, 1998a,b). Some have put forward the idea that odorants inhibit each other through competitive mechanisms at the olfactory receptor cells; thus the spatial code needed for odor identification may be lost in complex mixtures (Jinks and Laing, 1999).

We aimed to investigate odor mixture perception closer by using functional magnetic resonance imaging (fMRI) to record brain activation of subjects smelling odor mixtures. To do so, we wanted to take into account that the sense of smell exhibits a large variability in the population (Menashe et al., 2007). Even the simplest of tasks, such as determination of the lowest concentration needed to perceive an odor—the detection threshold—reveal huge variations between subjects. For instance, thresholds for androstadienone and phenyl ethyl alcohol (PEA) in 100 healthy young subjects—which interestingly were not correlated to each other—ranged over 12 logarithmic steps, or 4 orders of magnitude (Lundstrom et al., 2003). Moreover, androstadienone thresholds were bimodal in distribution; the two modes were separated by a 32 fold increase of concentrations. Extreme cases of a bimodal distribution can be observed for several odorants, which a large percentage of the general population cannot perceive at all, a state termed “specific anosmia” [e.g., androstadienone (Keller et al., 2007; Frasnelli et al., 2011) and androstenone (Boyle et al., 2006; Keller et al., 2007)]. One of the odorants for which a large percentage of the population exhibits either high or low sensitivity is ambroxan (AMB, with ~20% of the population exhibiting a low sensitivity—personal communication, Ursula Huchel), a synthetic compound belonging to the tetranorlabdane oxide class, which is widely used in perfumes.

We investigated inter-individual differences in mixture processing by comparing brain responses to odorous stimuli in two groups of subjects. Both groups were stimulated with (a) a single odorant for which high numbers of people exhibit either high or low sensitivity (AMB), (b) a complex mixture of several odorants, and (c) a mixture of (a and b). Both subject groups had similar general olfactory function, as assessed with a standardized olfactory test. However, one group was relatively insensitive (INS) to the single odorant, whereas the other group was relatively sensitive (SEN) to the same odor.

We had three specific hypotheses: firstly, (1) we expected that the SEN group, but not the INS group, would show measurable responses toward the single odorant. Secondly, we hypothesized that (2) the odor mixture would evoke similar activation patterns in both subject groups. Thirdly, we expected (3) the combination of the mixture with the single odorant to reveal larger activations in the SEN group than in the INS group.

On the practical side the current study was meant to investigate whether FMRI can be used to detect possible subliminal effects of odors on odor mixtures. Here it is important to say that FMRI has already been shown to indicate subliminal effects of odors on brain activation (e.g., Sobel et al., 1999). If this was possible then FMRI could be used in the future, for example to screen perfumes for “necessary” and “unnecessary” compounds which may or may not contribute to the overall effect of an odor on brain activation.

## Materials and methods

### Participants

The study was approved by the Ethics Board of the Technical University of Dresden Medical School (EK40022009). All subjects provided written informed consent. Prior to the study we had screened 58 subjects for their sensitivity to ambroxan (AMB). AMB is described as warm, slightly woody and voluminous (personal communication, Ursula Huchel). More importantly, many people exhibit either a high or a low sensitivity toward AMB (personal communication, Ursula Huchel). We diluted AMB in propylene glycol (Sigma, Germany) in a geometric series (1:10) starting at a 10% dilution. Thresholds were established in a paired comparison test; starting from the lowest concentration (concentration step 6; or 0.0001%), where AMB was presented together with a blank; and the subjects' task was to identify the bottle containing AMB. If subjects failed to identify the correct bottle, the concentration was increased, until subjects successfully performed the task three consecutive times. The concentration used was an estimate of AMB threshold. Our aim was to afterwards use AMB in a concentration which was below threshold for one group but above threshold for the other group. We opted for a concentration of 0.1% AMB, and therefore included only subjects whose threshold was either above or below that value. We therefore considered subjects with an AMB threshold of 2 (equaling 1% AMB) as insensitive (INS); subjects with an AMB threshold of 4 (equaling 0.01% AMB) and more were considered as sensitive (SEN). We invited 10 subjects of each group to participate in the scanning session.

This test had the tendency to underestimate the number of INS. In other words, if in the AMB threshold test a participant guessed correctly three times in a row he or she was labeled as SEN. The INS group, however, was not able to distinguish AMB from a blank three consecutive times on at least 6 trials, which makes us confident that they did indeed not perceive AMB.

In the INS group [average age: 23.2 (standard deviation: ±3.8) years] we included 8 mens and 2 womens (3 smokers), in the SENS group [26.3 (±5.3) years] the ratio was 4:6 (2 smokers). The difference in sex ratio (Fisher's exact test), smoker: non-smoker ratio (Fisher's exact test) or age (*t*-test) was not significant. We excluded subjects with a known history of neurological disorders, common cold and other states which may interfere with olfactory function, as well as subjects with a known olfactory dysfunction. Further, we excluded subjects with contraindications for a MRI examination. In order to determine normal olfactory function (and to exclude subjects with general hyposmia), we assessed detection thresholds for phenyl ethanol (PEA) and odor identification in all subjects using the Sniffin' Sticks test battery (Hummel et al., 1997).

### Olfactory testing

First, we assessed olfactory threshold to PEA in all subjects with a staircase method. On a given trial, subjects were presented with the odorant and with two blanks, in pen-like odor dispensing devices; their task was to identify the odorant. The odors were presented in a geometric series (1:2) of sixteen dilutions starting from 4% PEA dissolved in distilled water. Testing started at the lowest concentration. Concentrations were increased until correct detection occurred on two consecutive trials; then the staircase was reversed and moved downward. Threshold was defined as the mean of the last four out of seven staircase reversal points. We then tested the subjects' ability to identify 16 odors. Subjects were presented, together with the odor, with four cues, one being the correct answer. We counted the number of correct responses. After this procedure, we excluded one subject with general hyposmia (as indicated by an abnormally high threshold to PEA); thus, a total of 19 subjects were included in the analysis.

### Stimuli

Subjects were tested with three different odors and an odorless control stimulus. They were exposed to either 0.125% ambroxan (CAS# 6790-58-5; Henkel, Germany) in propylene glycol (CAS# 57-55-6; Sigma, Germany) (AMB), a 0.05% mixture of several odorants [consisting in equal parts of (a) 20% citronellol (CAS# 106-22-9), (b) 20% geraniol (CAS# 106-24-1), (c) 20% 2-phenyl ethanol (CAS# 60-12-8), (d) 5% 1-(1,2,3,4,5,6,7,8-Octahydro-2,3,8,8-tetramethyl-2-naphthyl)ethan-1-on (CAS# 54464-57-2), (e) 1% nerol (CAS# 106-25-2), and (f) 1% eugenol (CAS# 97-53-0) (all odors from Henkel, Germany)] in odorless propylene glycol (MIX), and a mixture of both (MIX + AMB). The concentrations of the single components were selected in order to be roughly iso-intense, as determined in a pilot experiment. We selected these odorants because they are frequently used in scented products and thus should be common to most participants. Odors were presented in a liquid mixture. In the scanner, odorless propylene glycol served as a control stimulus (CON).

### Procedure

Subjects were tested in one session of ~1.5 h. After they received detailed information about the study, they filled out questionnaires [handedness inventory (only right handed participants were included), self-rating of olfactory function]. We then performed olfactory threshold and identification tests as outlined above. We next assessed subjects' ability to distinguish between MIX and MIX + AMB using an oddball paradigm (Laska et al., 1997). Subjects were presented with three bottles containing MIX or MIX + AMB. In each triplet at least one bottle contained one of the two mixtures (e.g., bottle 1: MIX; bottle 2: MIX; bottle 3: MIX + AMB), in a randomized fashion. The partcicipants' task was to identify the bottle containing the odd odorant. We counted the total number of correct discriminations in nine repetitions. Subjects were then tested in the MR scanner, which lasted ~45 min including a total of 4 functional runs as well as an anatomical scan.

In each functional scan, one of the odor stimuli (AMB, MIX, or MIX + AMB) or CON was used. Subjects were instructed to passively smell the odors and to breathe normally; after each run they were asked to rate the delivered odor. Specifically, subjects were asked to verbally rate each odor on four dimensions (intensity, pleasantness, familiarity, and reward) using an 11 point scale, from 0 to 10. Zero indicated a very weak (very unfamiliar, very weakly rewarding) odor, whereas 10 indicated a very strong (very familiar, very rewarding) odor. For pleasantness, the scale ranged from −5 (very unpleasant) to 5 (very pleasant). In this context is worth noting that pleasantness and reward are related but distinct dimensions of odor perception (Small et al., 2001).

The anatomical scan lasted 15 min, whereas each of the functional runs lasted 5 min. Subjects were tested in a block design; during each functional run they were exposed to six “on”-blocks and six “off”-blocks in a pseudorandomized order. Each of the twelve blocks lasted 25 s. During the “on”-blocks odorized air was delivered to both nostrils intermittently (1 s odorized air; followed by 2 s no air; this was repeated 8 times, the block ended with a 1 s stimulation), with a flow of 2 L/min. Odorized air was delivered independent from the respiratory cycle. During the “off”-block, subjects received no stimulation. For odor delivery we used a custom-built device (Sommer et al., 2012), which allows for stimulation of the subject with odor enriched air via Teflon tubings; a constant air flow was delivered to either the subject, after being enriched with the odor in a small glass bottle, or to the outside of the scanner room, in case the subject was not stimulated; the lines for the different odors were completely separated; switching between conditions (odor, no odor; between different odorants) was controlled by a computer. After each functional run, subjects indicated perceived intensity, familiarity, pleasantness, and reward on an 11 point scale ranging from 0 to 10, as previously discussed. We measured the four dimensions in order to evaluate whether an additional component changed the perception of the mixture in any way.

### MRI scanning

We used a Siemens-Sonata 1.5 T scanner (Siemens, Erlangen, Germany) for data acquisition. For functional imaging, a spin echo/echo planar imaging sequence (epfid2d1.64; ep2d.max.bold protocol) was applied using software version syngo MR 2002B 4VA21A, with echo time (*TE*) = 35 ms, repetition time (*TR*) = 3000 ms, flip angle = 90°, and 1 average. For anatomical overlays, a T1-weighted (turboflash sequence) axial scan with 224 slices, voxel size of 1.6^*^1.1^*^1.5 mm, a repetition time (TR) of 3000 ms, echo time (TE) of 3.93 ms, and 2 averages (2130/3.93/2) was acquired.

### Data analysis

Psychophysical data was analyzed by means of SPSS 16.0 for Windows (SPSS Inc, Chicago, IL, USA); we computed *t*-tests to compare INS and SEN. The MRI data was analyzed by means of SPM8 (Wellcome Trust) implemented in Matlab (Mathworks, Natick, MS). Functional data were registered; motion corrected, and resliced using SPM8 pre-processing procedures. The resulting images were co-registered to the corresponding T1 volumes. We performed the analysis on images that were spatially normalized stereotactically transformed into MNI ICBM152-space; MNI-template supplied by SPM8) and smoothed [a 8 mm full width at half maximum (FWHM) Gaussian kernel]. As a second level analysis, we computed a factorial design with odor (4 levels: CON, AMB, MIX, MIX + AMB) as a within subject factor. We then contrasted resulting images using a paired sample *t*-test to highlight the difference between conditions and effects and two-sample *t*-tests for between group analyses. For within group comparisons (e.g., odor stimulation vs. no odor stimulation in all subjects) we corrected for whole brain family-wise error thresholding at *p* < 0.05 (indicated as “corrected”). For between group comparisons, (e.g., odor stimulation in INS vs. odor stimulation in SEN) we lowered this criterion to an uncorrected threshold of *p* < 0.001 with a cluster criterion of five voxels (indicated as “uncorrected”). Brain areas were labeled using the Mai atlas (Mai et al., 2008).

## Results

### Psychophysical data

**Figure 1:**
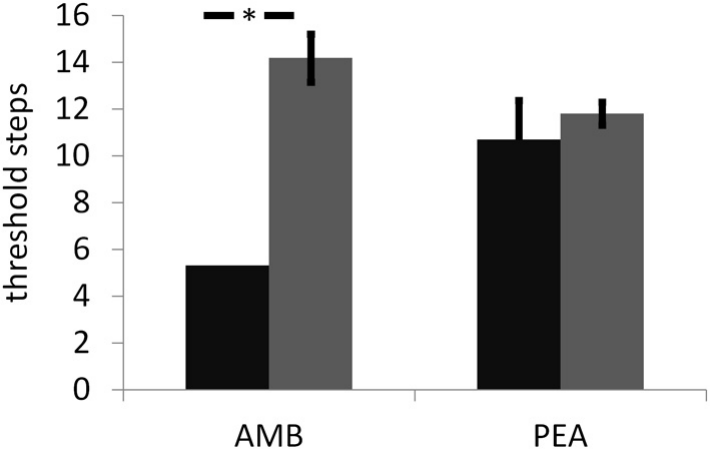
**Results for the threshold assessment (in log^2^ of the stock solution) for ambroxan (AMB) and the control odor phenyl ethyl alcohol (PEA) and in both groups of subjects (black bar: subjects insensitive to ambroxan; gray bar: subjects sensitive to ambroxan).** Error bars indicate standard errors. Asterisk indicates a significant difference between subject groups for AMB; no difference was observed for PEA.

The thresholds for PEA (INS: 10.7 [1.7]; SEN: 11.8 [±0.5]; n.s.; Figure 1) and identification scores (INS: 12.7 [±1.2] of 16; SEN: 13.7 [±1.2] of 16; n.s.) were not significantly different between groups. Although exhibiting a different sensitivity to AMB, both groups performed similarly when discriminating between MIX and MIX + AMB (INS: 4.0 [±0.4] of 9; SEN: 3.6 [±0.9] of 9; n.s.). In fact no subject in either group was able to distinguish the odors above chance levels. Additionally, with the exception of the familiarity of MIX + AMB, which was significantly more familiar for SEN than for INS (*p* = 0.016, uncorrected), *t*-tests did not reveal any significant difference between the two groups for the ratings of any odor obtained in the scanner (Table 1).

**Table 1:** **Subjective evaluation of odors in the scanner**.

**Odor**	**Dimension**	**INS average (standard deviation)**	**SEN average (standard deviation)**	***p* (*t*-test); uncorrected**
AMB	Hedonic	0.1 (2.6)	1.1 (3.2)	0.47
	Intensity	3.0 (2.7)	3.1 (3.6)	0.95
	Familiarity	2.1 (2.8)	3.8 (4.1)	0.31
	Reward	1.6 (2.2)	1.6 (3.8)	0.98
MIX	Hedonic	0.8 (1.9)	2.1 (2.0)	0.16
	Intensity	2.8 (3.4)	2.5 (3.1)	0.86
	Familiarity	2.0 (3.1)	3.7 (3.2)	0.26
	Reward	2.1 (2.9)	2.8 (2.4)	0.59
MIX + AMB	Hedonic	0.1 (1.7)	2.1 (2.5)	0.06
	Intensity	2.9 (2.4)	3.0 (2.5)	0.92
	Familiarity	1.0 (2.5)	4.2 (2.7)	0.016
	Reward	0.9 (2.6)	3.2 (2.5)	0.06

### Functional MRI data

**Figure 2:**
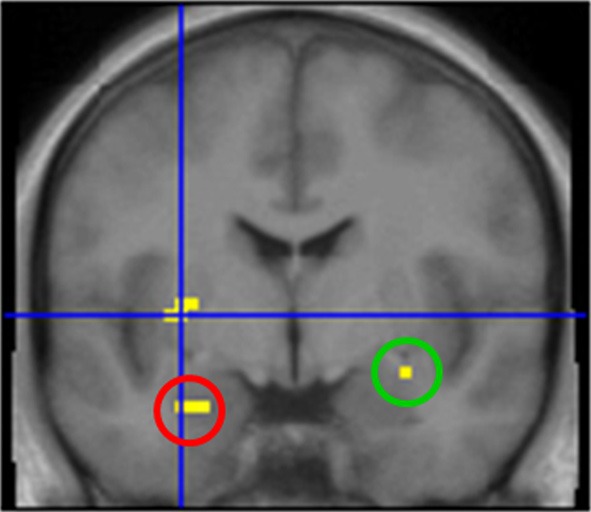
**Brain activation after stimulation with odors.** Highlighted areas include left insula (cross hair), left amygdala (red circle) and right amygdala/piriform cortex (green circle). Contrast: [AMB + HEN + MIX] vs. CON; *y* = −1.

First, we grouped all odor conditions in all subjects and compared them to baseline activation (AMB + MIX + AMB + MIX vs. CON). Here we observed activations of chemosensory processing brain regions, such as left insula, bilateral amygdala, and piriform cortex (Table 2 for summary of brain activations; Figure 2).

**Table 2:** **Brain activations following odor stimulation in all subjects; contrast: all odors vs. baseline (AMB + HEN + MIX) − CON (*p* < 0.05; corrected)**.

	***X***	***Y***	***Z***	***p* (cluster)**	**Voxels**	**Peaks**	**Structure**
1	−36	2	−35	<0.001	10	1	L inferior temporal G
2	−27	8	10	<0.001	44	2	L insula
3	39	−25	46	<0.01	3	1	R postcentral G
4	−39	−13	16	<0.01	8	3	L insula
5	−24	−4	−23	<0.05	7	1	L amygdala
6	21	−25	37	<0.05	3	1	R cingulate G
7	−33	−7	13	<0.05	2	1	L insula
8	30	−4	−14	<0.05	1	1	R amygdala + piriform C

**Figure 3:**
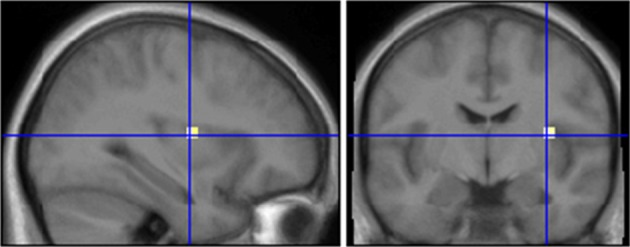
**Comparison of subjects who smell ambroxan and subjects who don't after stimulation with ambroxan.** Area in cross hairs: right insula. Contrast SEN[AMB] vs. INS[AMB]; *x* = 33; *y* = −7.

We next analyzed the differences between INS and SEN for the odor AMB. We computed contrasts between both subject groups while they were presented with AMB (SEN [AMB] vs. INS[AMB]). We observed SEN to exhibit larger activations in chemosensory processing areas (insula) as well as other brain regions than INS (Table 3; Figure 3).

**Table 3:** **Specific brain activations following ambroxan stimulation between subjects who perceive ambroxan (SEN) and those who don't (INS); contrast: AMB [SEN] vs. AMB [INS] (*p* < 0.001; uncorrected)**.

***X***	***Y***	***Z***	***T* (peak)**	**Voxels**	**Structure**
−24	26	7	4.1	9	L insula
33	−7	10	4.1	6	R insula
−3	−1	37	4.1	23	L cingulate
−36	17	10	3.8	11	L insula
−24	−10	−11	3.5	5	L parahippocampal G

**Figure 4:**
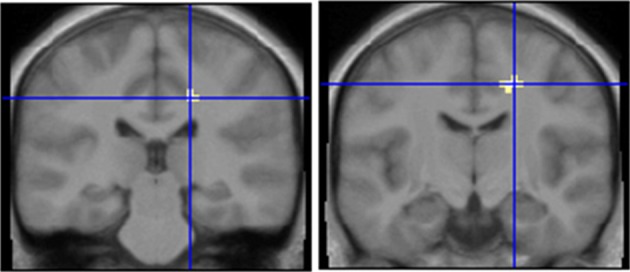
**Comparison of an ambroxan containing mixture with a mixture which does not contain ambroxan in subjects who perceive ambroxan (left) and subjects who do not perceive ambroxan (right).** Area in cross hairs: right cingulate. Contrast left: SEN[MIX + AMB] vs. SEN[MIX] masked with [AMB + MIX+AMB + MIX] vs. CON; *y* = −28; Contrast right: INS[MIX + AMB] vs. INS[MIX] masked with [AMB + MIX+AMB + MIX] vs. CON; *y* = −13.

As a last step, we compared brain activations following stimulation with the odor mix which contained AMB (MIX + AMB) and the one without AMB (MIX). We performed this analysis in both groups separately. We further masked the results by the general contrast (ODORS vs. CON) in order to exclude false positive activations. In the SEN group [SEN (MIX+AMB vs. MIX)] we observed activations in the right inferior occipital cortex, the right striate, the right cingulate, the left precentral gyrus (Table 4). When performing the same contrast in INS [INS(MIX+AMB vs. MIX)], we obtained a similar activation in the right cingulate; no other brain region was significantly activated in this contrast (Table 5). For a comparison of both cingulate regions, see Figure 4. A direct masked comparison between these maps from both subject groups revealed activations in bilateral insula (on the right side stretching into the precentral gyrus, see Table 6).

**Table 4:** **Brain activation due to ambroxan within a mixture in ambroxan sensitive subjects; contrast: SEN: MIX + AMB vs. MIX (masked ALL vs. CON) (*p* < 0.001; uncorrected)**.

***X***	***Y***	***Z***	***T* (peak)**	**Voxels**	**Structure**
30	−85	−8	3.7	6	R inf occipital G
−39	−13	43	3.7	15	L precentral G
33	−61	10	3.7	10	R striate area
18	−28	37	3.6	6	R cingulate

**Table 5:** **Brain activation due to ambroxan within a mixture in ambroxan insensitive subjects; contrast: INS: MIX + AMB vs. MIX (masked ALL vs. CON) (*p* < 0.001; uncorrected)**.

***X***	***Y***	***Z***	***T* (peak)**	**Voxels**	**Structure**
21	−13	43	3.9	13	R cingulate

**Table 6:** **Brain activation due to ambroxan within a mixture in ambroxan; difference between sensitive and insensitive subjects; contrast: SEN(MIX + AMB) vs. MIX vs. INS(MIX + AMB vs. MIX) (masked ALL vs. CON) (*p* < 0.001; uncorrected)**.

***X***	***Y***	***Z***	***T* (peak)**	**Voxels**	**Structure**
48	−4	25	4.5	12	R insula and precentral G
−21	20	−5	3.9	6	L insula
−39	−4	13	3.6	9	L Insula

## Discussion

In this study we report four major findings.

First, we show that adding a perithreshold odorant to a mixture renders a new mixture which is very difficult to be distinguished from the original mixture. In the present study we used mixtures of 6 + 1 components. This result is in line with several studies which showed that human beings perform relatively poor when analyzing the components of complex mixtures. In a series of studies, humans were able to detect and identify the single components within a complex mixture of odors only if the latter consists of less than five odorants (Livermore and Laing, 1998a,b).

However, other researchers showed that humans can distinguish between complex mixtures of more than five components (Laska and Hudson, 1992; Sinding et al., 2013), especially if odorants are omitted. Researchers have thus put forward the idea of olfaction being a “synthetic” sense, similar to color vision and in contrast to gustation (Livermore and Laing, 1998a). A possible underlying neuroanatomical correlates may be the posterior piriform cortex which codes for odor quality, as opposed to the anterior piriform cortex, which is functionally located upstream and codes for chemical structure of the odorant (Gottfried et al., 2006). In general, our research therefore corroborates this body of literature as it shows that both, subjects who could perceive AMB when presented as a single compound and subjects who could not perceive AMB when presented as a single compound, performed similarly when trying to distinguish between two mixtures, AMB positive and AMB negative mixtures.

These results are also interesting with regard to the fact that familiarity of MIX + AMB differed between groups. Accordingly, the influence of familiarity in the discrimination of odor mixtures may be less pronounced than previously thought [Rabin MD (1988) Experience facilitates olfactory quality discrimination. Perception Psychophysics 44:532–540].

Secondly, with regards to brain activations a picture emerges which is in contrasts to the behavioral findings. When MIX + AMB was contrasted with MIX, the sensitive group showed activations of several brain regions including the right inferior occipital gyrus, the right striate area and the left precentral gyrus; unlike the insensitive group which did not exhibit any activation in these areas. To the best of our knowledge, this study is the first to show evidence for a broad sensitivity range for olfactory mixtures, similar to single substances (Lundstrom et al., 2003; Menashe et al., 2007). Interestingly, both, the INS and the SEN showed activation in the right cingulate cortex when contrasting MIX + AMB with MIX. Here we would like to remind the reader that the INS group did not perceive AMB (at least at the concentration we used) and they are not able to distinguish MIX + AMB from MIX; yet, this brain region is significantly more activated when exposed to MIX + AMB. The cingulate cortex plays a crucial role in odor mixture processing, as the left cingulate is activated stronger when subjects are presented with a binary mixture than with both single components separately (Boyle et al., 2009). One may hypothesize that, in analogy, the presence or absence of AMB in the concentration we used leads to a differential activation in the cingulate cortex regardless of whether the subjects could perceive the compound or not. In other words, this specific brain region reacts to the addition of a component, even in the absence of a perceivable difference.

These observations are particularly interesting if one considers several studies on mixtures involving subthreshold components: for example, when investigating perception thresholds for different mixtures, even components at subthreshold levels, i.e., in concentrations that were below the threshold when the substance was tested on its own, interacted with other mixture components suggesting hyperadditivity and enhancement (Laska and Hudson, 1991). Another study, on wine aromas, confirmed this finding. Here, adding a woody smelling odorant in a concentration at which on its own it could not perceived by participants, altered a fruity odorant, so that participants could distinguish between both stimuli (fruity vs. fruity + subthreshold woody) (Atanasova et al., 2005). Similarly, adding subthreshold concentrations of acetic acid or butyric acid increased detectability of a two component mixture significantly more likely (Miyazawa et al., 2008). Our observations may therefore provide a neurophysiological underpinning for these behavioral results. Interested researchers could investigate the activation patterns caused by adding components and the limits of these mechanisms in future studies.

Third, we show that subjects presented with an odor at sub-threshold concentrations show lesser activation in the insula than subjects for which the odor—at the same concentration—is above detection threshold. When sensitive subjects were presented with AMB, they exhibited larger activations than insensitive subjects in several olfactory processing brain regions, all of which are located in the left and right insula. The insula is prominently involved in olfactory processing—it is activated when subjects perform different olfactory tasks, ranging from passive stimulation to higher order olfactory tasks (Savic et al., 2000; Sobel et al., 2000; Bengtsson et al., 2001; Dade et al., 2002; Gottfried and Dolan, 2003; Wicker et al., 2003; Djordjevic et al., 2005; Wang et al., 2005; Hillert et al., 2007; Plailly et al., 2007). Our results are in line with these earlier findings and highlight the fact that the insula is involved in conscious and inconscious odor processing and/or odor perception.

Fourthly, we observed that different brain activations between subjects who perceived AMB and those who did not, when they were presented with the AMB containing mixture (MIX + AMB). Specifically, stronger activations in the cingulate cortex were observed in the SEN group compared to the INS group. The cingulate cortex is part of the pain matrix, and is therefore activated when subjects are exposed to trigeminal stimulation (Bensafi et al., 2008; Albrecht et al., 2010). In this current study, the odor mixture used contained components which are known to activate the trigeminal system, e.g., eugenol (Wise et al., 2012). It could consequently be interpreted that the larger activation in the SEN group may be caused by a stronger trigeminal perception of the mixture. On the contrary, behavioral results indicate that there was no group difference in perceived intensity.

Additionally, aside from being implicated with trigemial activity, earlier reports show that the cingulate cortex is also involved in the processing of odors. The cingulate was indeed activated when participants smelled a binary mixture compared to its single components (Boyle et al., 2009), or when subjects received combinations of taste and smell stimuli (Small et al., 2004). The current data may indicate a similar superadditive effect due to the perception of the more complex mixture leading to activation of the cingulate cortex. This hypothesis could be investigated in future studies.

Furthermore, we observed activation of occipital brain regions in the same group of subjects; however, the reason for this is currently unclear. One may speculate that the unconscious perception of AMB within the mixture triggered (visual) imagery in the SEN group (Bensafi et al., 2007); this was not the case in the INS group.

Due to time constraints we used a rather lenient but fast test when determining the AMB threshold. The main limitation in the study is based on the probability that some subjects may have been classified into the SEN group as they may have correctly identified the ambroxan odor by chance. The probability is 0.125 for a given concentration, leading to a probability of 0.375 that a given subject was classified as SEN although s/he did not perceive AMB at the concentration steps 4–6. Based on binomial statistics, there is a 55% chance that up to 4 subjects were classified as SEN although they were insensitive to the AMB concentrations. This may have caused a caveat in the interpretation of our results.

One additional aim of the current study was also to investigate whether FMRI can be used to detect possible subliminal effects of odors on odor mixtures. The current results suggest that this is possible. Thus, FMRI could be used in the future, for example to screen perfumes for (potentially very expensive) compounds which may or may not contribute to the overall effect of an odor on brain activation. In analogy, the expense for compounds not contributing to the overall effect might be saved.

## Conclusions

An odor presented within a mixture of odors can influence activation of brain regions such as the cingulate and the insula, even if subjects are not able to distinguish the mixture with and without the odor. This appears to be true even for subjects for which the odor, presented on its own, is too weak to be perceived. On the practical side these results suggest that the addition of a certain compound to a mixture of odors may not be detected on a cognitive level; however, this additional odor may significantly change the cerebral processing of this mixture.

### Conflict of interest statement

Dr. U. Huchel is an employee of Henkel, Germany, who sponsored the study. The other authors declare that the research was conducted in the absence of any commercial or financial relationships that could be construed as a potential conflict of interest.

## Abbreviations

PET: positron emission tomography
OFC: orbitofrontal cortex
PEA: phenyl ethyl alcohol
AMB: ambroxan
MIX: odor mixture consisting of six components
MIX+AMB: odor mixture consisting of MIX and AMB
SEN: subject group sensitive to AMB
INS: subject group insensitive to AMB
CON: odorless control stimulus (propylene glycol)
FWHM: full width at half maximum.
